# Pan-Cancer Metabolic Landscapes: A Multiomics View

**DOI:** 10.1021/acs.jproteome.6c00346

**Published:** 2026-08-27

**Authors:** Yiguang Bai, Qiaoling Chen, Yuan Li

**Affiliations:** † Department of Orthopedics, Beijing Anzhen Nanchong Hospital, Capital Medical University (Nanchong Central Hospital), Nanchong 637000, China; ‡ Department of Orthopedics, Nanchong Central Hospital, The Second Clinical Medical College of North Sichuan Medical College, Nanchong 637000, China; § Department of Oncology, Beijing Anzhen Nanchong Hospital, Capital Medical University (Nanchong Central Hospital), Nanchong 637000, China; ∥ Department of Oncology, Nanchong Central Hospital, The Second Clinical Medical College of North Sichuan Medical College, Nanchong 637000, China; ⊥ National Bioinformatics Infrastructure Sweden (NBIS), Science for Life Laboratory, 5193 Lund University, Lund 22387, Sweden; # Department of Immunotechnology, 5193 Lund University, Medicon Village, Lund 22387, Sweden

**Keywords:** pan-cancer, metabolism, multiomics integration, spatial, glycosylation, nucleotide, immunometabolism

## Abstract

Metabolic reprogramming fuels cancer progression, but whether common metabolic patterns exist across diverse malignancies remains incompletely understood. To address this, we integrated large-scale proteomic, transcriptomic [The Cancer Genome Atlas (TCGA)], and spatial transcriptomic (Spatial Meta-Transcriptome Database) data sets comprising 3226 samples across 24 human cancer types. Utilizing a highly controlled, predominantly patient-matched design to minimize background noise, we characterized the pan-cancer metabolic landscape via reaction-level functional task scoring, pathway enrichment, multiomics integration, network modeling, survival analysis, and microenvironmental coupling. Consistently upregulated glycan biosynthesis pathways—particularly fucosylation and sialylation—and broadly enhanced nucleotide metabolism highlighted conserved programs potentially associated with tumor growth, adaptation, and immune evasion. These signatures proved robust against variations in tumor purity across the TCGA cohorts. Furthermore, gene coexpression network modeling prioritized specific hub genes associated with this pan-cancer restructuring. Exploratory high-resolution mapping aligned tumor immunogenicity with specific glycan pathways. Notably, tumor-intrinsic glycan upregulation negatively correlates with their immune score alignment, suggesting that tumor hyperactivation may obscure immune signals in bulk tissue. Together, our results define a robust computational landscape of shared metabolic alterations, providing a data-driven framework to guide future experimental validation of candidate therapeutic targets across diverse oncological contexts.

## Graphical abstract

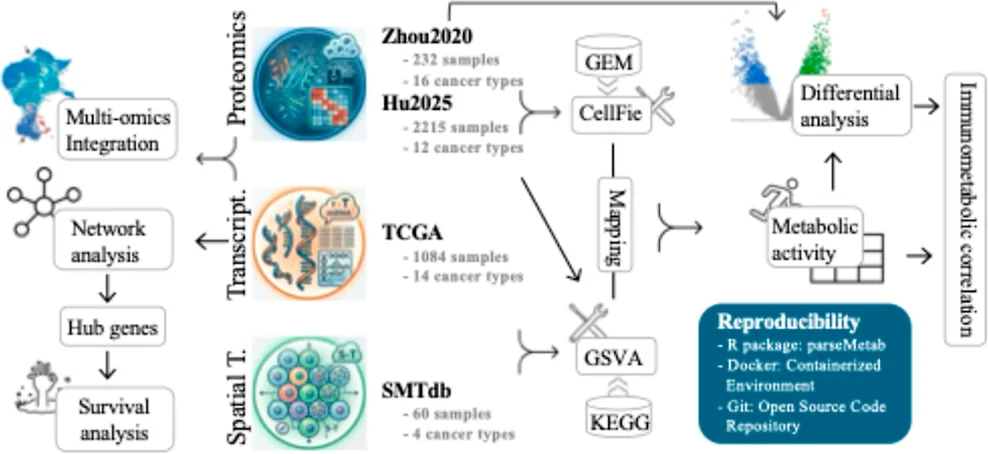

## Introduction

Metabolic reprogramming is a hallmark of cancer, enabling malignant cells to proliferate rapidly, survive stress, and adapt to dynamic microenvironments.^1–3^ Beyond altered energy production, cancer metabolism supports biosynthesis, redox balance, and signaling pathways that collectively sustain tumor growth.^4,5^ While central metabolic pathways have been extensively studied, key processes such as glycan biosynthesis—which play critical roles in protein modification, cell communication, and immune interactions—have received comparatively limited pan-cancer attention.^6^ This is notable given that glycan synthesis—a complex enzymatic process occurring within the endoplasmic reticulum and Golgi apparatus—adds regulatory layers that significantly influence tumor behavior.^7,8^ Although aberrant glycosylation is broadly recognized as a hallmark of tumorigenesis affecting essential cancer cell functions, its prevalence and variation across diverse tumor types remain incompletely characterized in systematic pan-cancer studies.^9^

Many studies of cancer metabolism focus on individual tumor types.^1–3^ Pan-cancer analyses, when performed, are frequently based on a single omics layer—most commonly transcriptomic data from the Cancer Genome Atlas (TCGA).^10–12^ Multiomics pan-cancer approaches often emphasize selected metabolic pathways/genes rather than systematically characterizing the full metabolic landscape, or they focus broadly on cross-modality integration within TCGA data layers.^13–16^ Consequently, while these previous pan-cancer metabolic studies have successfully mapped broad oncogenic hallmarks, they frequently lacked the resolution to trace specific reaction trajectories or decouple tumor-intrinsic wiring from surrounding microenvironmental noise. To address these limitations, we developed an integrative computational framework that couples large-scale proteomic, bulk transcriptomic, and spatial transcriptomic data sets across 3226 tissue samples. Rather than solely relying on generic gene-set enrichment tools, our pipeline also deploys reaction-level functional task scoring, coexpression network modeling, survival analysis, and tumor purity constraints to systematically isolate tumor-specific metabolic dependencies from microenvironmental confounding. Furthermore, by leveraging spatial data to resolve distinct tissue compartments and implementing immunometabolic coupling, our exploratory analysis highlights a potentially localized, contextualized view of tumor metabolism. Ultimately, this framework maps shared metabolic programs and prioritizes pathways that may represent candidate therapeutic targets for future experimental validation across diverse cancer types.

## Materials and Methods

To study pan-cancer metabolic reprogramming, we compared tumor and matched nontumor samples across multiomics data sets, integrating two proteomics resources (Zhou2020, Hu2025)^17,18^ alongside the TCGA transcriptomics cohorts^12^ and the Spatial Meta-Transcriptome Database (SMTdb) collection.^19^ For bulk data sets, tumor samples were compared to matched nontumor tissues, while for spatial transcriptomics data, comparisons were conducted between malignant spots and stromal spots within the same tissue section. For each SMTdb tissue section, raw transcript counts from all malignant and stromal spots were aggregated separately to generate pseudobulk RNA-seq profiles. The majority of analyzed samples were matched tumor–normal pairs from the same patients, enabling robust intrapatient comparisons across diverse cancer contexts (Table S1).

We used the Zhou2020 data set in its preprocessed form as provided by the original publication,^17^ while the Hu2025 collection was curated and uniformly reprocessed in the original study.^18^ Transcriptomic data for multiple cancer types were obtained from TCGA^12^ using TCGAbiolinks v2.34.1.^20^ Raw gene-level counts generated using Spliced Transcripts Alignment to a Reference (STAR) were retrieved, including only primary tumor and solid tissue normal samples with paired data per subject. Genes were filtered to retain those with counts of >5 in at least 15% of samples per group or at least two samples when the 15% threshold corresponded to fewer than two samples. Data were normalized using the limma-voom pipeline (limma v3.62.1,^21^ edgeR v4.4.0 ^22^), yielding log2-transformed counts per million (logCPM). Spatial transcriptomics data for multiple cancer types were obtained from SMTdb.^19^ For each cancer type, expression data per spot and spatial annotations (malignant, stromal, boundary) were retrieved from the SMTdb website. We included only cancer types with at least three slices containing malignant spots and three slices with stromal spots. For each slice, raw counts from malignant and stromal spots were aggregated separately into pseudobulk profiles, typically yielding one malignant and one stromal sample per slice. Genes were filtered using the same criteria as those for the TCGA data. Pseudobulk data were normalized with edgeR (v4.4.0) using the trimmed mean of *M*-values with singleton pairing (TMMwsp) normalization method, which accounts for sample composition bias and variable expression across distinct cell populations, resulting in logCPM values.

### Metabolic Task Scoring Using CellFie

To interpret proteomics data across 16 cancer types from the Hu2025 and Zhou2020 data sets, proteins were grouped into metabolic tasks, and task activities were quantified using the Cellular Functions Inference (CellFie) tool on the GenePattern Web server.^23^ Metabolic tasks represent small reaction modules converting specific substrates to products, reflecting metabolic reprogramming often associated with tumor growth and therapy resistance.^2,3^

Proteins were mapped to Entrez Gene IDs using org.Hs.eg.db v3.20.0 ^24^ and AnnotationDbi v1.68.0.^25^ The Zhou2020 data set was log_2_-transformed prior to analysis, while the Hu2025 data set was converted to a linear scale via exponentiation to resolve negative values and align with input requirements. Feature activity was determined via local thresholding (local).Threshold.Type = “minmaxmean”), with thresholds set as feature means constrained between the 25th and 75th percentiles of the overall abundance.

All Zhou2020 samples were analyzed together, whereas the Hu2025 data set was stratified by cancer type and submitted in independent batches. CellFie categorized metabolic tasks into seven classes (lipid, energy, nucleotide, amino acid, glycan, carbohydrate, and vitamin and cofactor metabolism). To align with Kyoto Encyclopedia of Genes and Genomes (KEGG)^26^ classifications, several tasks originally assigned to carbohydrate metabolism were reassigned to glycan metabolism: synthesis of lactose, uridine diphosphate (UDP)-glucose synthesis, UDP-galactose synthesis, UDP-glucuronate synthesis, guanosine diphosphate (GDP)-l-fucose synthesis, GDP-mannose synthesis, UDP-*N*-acetyl- *d* -galactosamine synthesis, cytidine monophosphate (CMP)-*N*-acetylneuraminate synthesis, *N*-acetylglucosamine synthesis, glucuronate synthesis (via inositol), and glucuronate synthesis (via UDP-glucose).

### Metabolic Enrichment Scoring Using KEGG-Based GSVA

To infer metabolic activity from the two aforementioned proteomics data sets, as well as the TCGA transcriptomic and SMTdb pseudobulk RNA-seq data, metabolic gene sets and pathways along with their classes were extracted from KEGG using KEGGREST v1.46.0.^27^ Gene Set Variation Analysis (GSVA) was performed on the metabolic gene sets (size 5–500) to calculate pathway-level enrichment scores reflecting relative metabolic activity per sample. GSVA, a nonparametric unsupervised method, estimates variation in pathway activity across samples by integrating gene expression into pathway-centric scores, enabling sample-wise comparisons. Analysis was conducted using GSVA v2.0.7.^28^

### Cross-Workflow Metabolic Profile Correlation Analysis

To evaluate the consistency of metabolic state estimations across distinct computational workflows—specifically CellFie and GSVA—pairwise Spearman rank correlation coefficients ρ were calculated using normal tissue samples from two independent data sets, Zhou2020 and Hu2025, independently for each tissue type using overlapping sample profiles. To isolate robust metabolic relationships transcending individual tissue variations, the element-wise median ρ was calculated across all tissue types for each data set collection, retaining only pairs with a high-stringency correlation of median ρ > 0.5. The filtered correlation structures from both data set collections were subsequently merged and consolidated into a unified network of top-correlated hits by independently extracting the single strongest correlated KEGG pathway for each distinct CellFie task and the single strongest correlated CellFie task for each unique KEGG pathway, removing duplicate overlapping links to minimize visual complexity. Hierarchical parent-level classifications were then appended to frame these links within a four-tier biological network mapping: CellFie Systems (Tier 1) → CellFie Tasks (Tier 2) → KEGG Pathways (Tier 3) → KEGG Classes (Tier 4). Finally, this integrated layout was visualized via an interactive multilayer Sankey diagram using plotly v4.12.0.^29^

### Differential Analysis of Metabolic Activities between Cancer and Normal Samples

To understand how cancer cells reprogram their metabolic processes to support rapid growth, survival, invasion, and therapy resistance, we performed univariate differential analysis using linear models implemented in limma v3.62.2.^21^ Each metabolic task or pathway was tested individually for differential activity between the tumor and matched normal samples. In this analysis, each cancer type was analyzed separately using Zhou2020_CellFie, Hu2025_CellFie, Zhou2020_KEGG, Hu2025_KEGG, TCGA_KEGG, and SMTdb_KEGG data.

Differential analysis was only performed on cancer types with both cancer and normal samples, requiring a minimum of two tumor-normal pairs. For these cohorts, paired differential analysis was conducted using limma v.3.62.1 on each cancer individually. The correlation between paired tumor and matched normal samples from the same patient was modeled using the duplicateCorrelation­() function, allowing for appropriate handling of intrapatient dependencies. Differential testing was performed using limma’s empirical Bayes (EB) moderation, which stabilizes variance estimates and improves statistical power, particularly in data sets with limited sample sizes. To further increase robustness, the EB procedure was carried out with robust = TRUE to reduce the influence of potential outliers and trend = TRUE to account for mean–variance relationships in the metabolic activity data. For the Zhou2020_CellFie and Hu2025_CellFie data, standard unpaired limma differential analysis was performed wherever there was not enough paired data. In the Zhou2020_CellFie and Hu2025_CellFie data sets, CellFie task metabolic activity scores were log2-transformed before limma analysis, and only metabolic tasks with at least two quantified scores in each sample group (normal and tumor) were included in limma analysis. For the 12 cancer types in the Hu2025_CellFie data, we also controlled for batch effects when multiple batches were present within a single cancer type. *p*-values were adjusted using the Benjamini–Hochberg correction to control the false discovery rate.

Bulk tumor samples, such as those in the TCGA data set, represent a complex mixture of malignant cells, normal cells, blood vessels, stroma, and infiltrating immune cells. To evaluate whether tumor-associated transcriptomic alterations were robust against variations in tumor purity, we performed a sensitivity analysis using TCGA_KEGG data. Paired differential enrichment was re-evaluated by adjusting for tumor purity, which was quantified via Consensus Purity Estimation (CPE).^30^ Matched normal samples were assigned a CPE score of 0, and only pairs with available tumor CPE scores were included. Both TCGA transcriptomic and purity data were retrieved using the R package TCGAbiolinks v2.34.1.^20^

### Immunometabolic Characterization and Coupling Analysis

To map robust interactions between tumor metabolism and microenvironment shifts, TCGA sample-matched cellular immune infiltration scores were downloaded via the ESTIMATE platform.^31^ Nonparametric Spearman’s rank correlation analysis was executed independently within each TCGA cancer type to evaluate the baseline association (immunometabolism) between sample-level KEGG metabolic pathway GSVA enrichment profiles and continuous ESTIMATE immune scores.

To evaluate whether tumor-specific metabolic pathway dysregulation is systematically linked to host immune tracking across distinct malignancies, a secondary cross-cohort analysis was performed (immunometabolic coupling). For each filtered KEGG metabolic pathway, a Spearman rank correlation was calculated across distinct TCGA cohorts to evaluate the direct relationship between the cohort-level differential activity effect size—quantified as *t*-values derived via linear modeling (limma)^21^ comparing tumor to matched normal controls—and the corresponding intratumor immunometabolism metrics (ρ). The resulting pan-cancer immunometabolic correlation matrix was visualized as a dot plot using ggplot2 v4.0.2.^32^

### Differential Analysis of Metabolic-Related Protein Abundance between Cancer and Normal Samples

CellFie was originally designed for bulk RNA-seq with extensive gene coverage; thus, some metabolic tasks in proteomics data may have insufficient protein measurements for stable activity scores. To evaluate the consistency of CellFie-derived patterns, we performed paired differential expression analysis for each metabolic class at the protein level by using the same limma-based approach. This evaluation included only paired samples and proteins that were quantified in at least two replicates per group (normal and tumor).

### Gene Coexpression Network Analysis

Gene coexpression network analysis was performed to uncover coordinated regulation and identify computationally prioritized hub genes associated with metabolic rewiring in cancer. For each KEGG metabolic class, pairwise Spearman correlations among member genes were computed across TCGA samples within each cancer type and averaged across all cancer types to generate a unified pan-cancer coexpression matrix. To transition from a raw correlation matrix to a weighted network, the absolute correlation coefficients were raised to an exponential soft-thresholding power parameter, denoted as β. Connectivity was subsequently calculated as the row sums of the resulting weighted adjacency matrix, excluding self-correlations.

Mathematically, applying the exponential power acts as a continuous, nonlinear filter that disproportionately suppresses weak background correlations and minor biological noise toward zero while preserving and highlighting strong, high-confidence functional relationships.

To reduce manual bias in selecting this parameter, we implemented an optimization algorithm that evaluated β values from 1 to 20. The pipeline automatically selected the lowest integer β that forced the network to satisfy a scale-free topology fit index (*R* ^2^ ≥ 0.8). This index is a linear regression metric evaluating how closely the network’s connectivity distribution follows a power-law distribution, which is characteristic of many natural biological networks, wherein a tiny fraction of core components are highly connected. Achieving this threshold indicates that the network layout has aligned with a structured, biological topology rather than random background noise. This optimized structure provides the necessary statistical resolution to identify candidate regulators (hubs) based on network connectivity, which was enforced by applying a stringent top 1% connectivity threshold. Finally, to evaluate the mathematical robustness of this network infrastructure, a formal sensitivity analysis was conducted by repeating the entire pipeline across three distinct scale-free topology thresholds (*R* ^2^ ≥ 0.7, 0.8, and 0.9) simultaneously with five connectivity cutoffs (0.01, 0.05, 0.1, 0.2, and 0.3). Edges with an absolute mean correlation >0.6 were retained to construct undirected weighted networks using igraph v2.0.3.

### Survival Analysis: Multivariate Cox Proportional Hazards Regression and Multigene Signature Construction

Multivariate Cox proportional hazards regression was performed to evaluate the independent prognostic value of six hub genes, encoding alpha-1,3-mannosyltransferase (ALG3), ribophorin I (RPN1), ribophorin II (RPN2), deoxythymidylate kinase (DTYMK), thymidine kinase 1 (TK1), and ribonucleotide reductase regulatory subunit M2 (RRM2). To handle clinical metadata variations programmatically, a dynamic formula builder was used to adjust for available confounding factors (age, disease stage, and gender). Models were constructed using the R package survival v3.8–3,^33^ assessing individual gene expression levels alongside existing clinical confounders as independent predictors of overall survival. To prevent artifacts from severe data sparsity or multicollinearity, models that failed to converge or exhibited mathematical instability—defined as an excessively large standard error (SE > 5)—were excluded from downstream visualization.

To evaluate the collective clinical relevance and complementary prognostic value of these central network features relative to individual markers, a multigene composite risk signature was constructed. For each cohort, a multivariate Cox proportional hazards model incorporated the expression profiles of all hub genes simultaneously for each KEGG metabolic class, adjusted for available clinical covariates (age, stage, and gender). To isolate the independent prognostic contribution of the genetic architecture from clinical factors, a multigenes risk score was calculated for each patient via matrix multiplication of the adjusted hub-gene coefficients from the model by their corresponding expression matrices, thereby mathematically excluding clinical covariate values from the final score summation. Finally, univariate Cox regression was performed to evaluate the prognostic values of these isolated genetic risk scores.

### Multiomics Integration

To integrate the two proteomics data sets with the TCGA cohorts, we employed the integrative nonnegative matrix factorization (iNMF) method from rliger v1.0.0 ^34^ for a joint analysis of KEGG-derived metabolic activity scores. The analysis was restricted to pathways associated with glycan and nucleotide metabolisms. To quantitatively evaluate integration performance and batch correction, a Permutational Multivariate Analysis of Variance (PERMANOVA)^35^ was performed using the adonis2­() function from the R package vegan v2.7–5.^36^ The test utilized a Euclidean distance matrix derived from the aligned iNMF factor coordinates (H.norm). Variance partitioning was calculated sequentially, controlling for data set batch effects prior to evaluating the biological condition (tumor vs normal), utilizing 999 permutations.

## Results and Discussion

To characterize pan-cancer metabolic reprogramming, tumor and matched nontumor samples were analyzed across independent proteomics, bulk transcriptomics, and spatial transcriptomics data sets, encompassing 24 cancer types and 3226 samples (1610 tumor, 1616 nontumor) from multiple studies (Figure 1A, Table S1). Metabolic task activities were first inferred using the CellFie framework on two proteomics data sets: the unified pan-cancer resource by Zhou et al. 2020 ^17^ and a curated proteomics collection by Hu et al. 2025,^18^ identifying 176 and 138 active tasks, respectively, grouped into seven metabolic classes comprising 77–136 proteins (Figure 1B, S1O–P; Table S1). Complementarily, GSVA^28^ analysis was performed using KEGG.^26^ GSVA was applied to the aforementioned proteomics data sets, transcriptomic data from TCGA,^12^ and spatial transcriptomic pseudobulk RNA-seq data from SMTdb.^19^ This analysis resolved 55–83 metabolic pathways across 11 classes, representing 1454–1705 genes (Figure 1B, S1Q-S, and S1M-V; Tables S1, S2).

**Figure 1:**
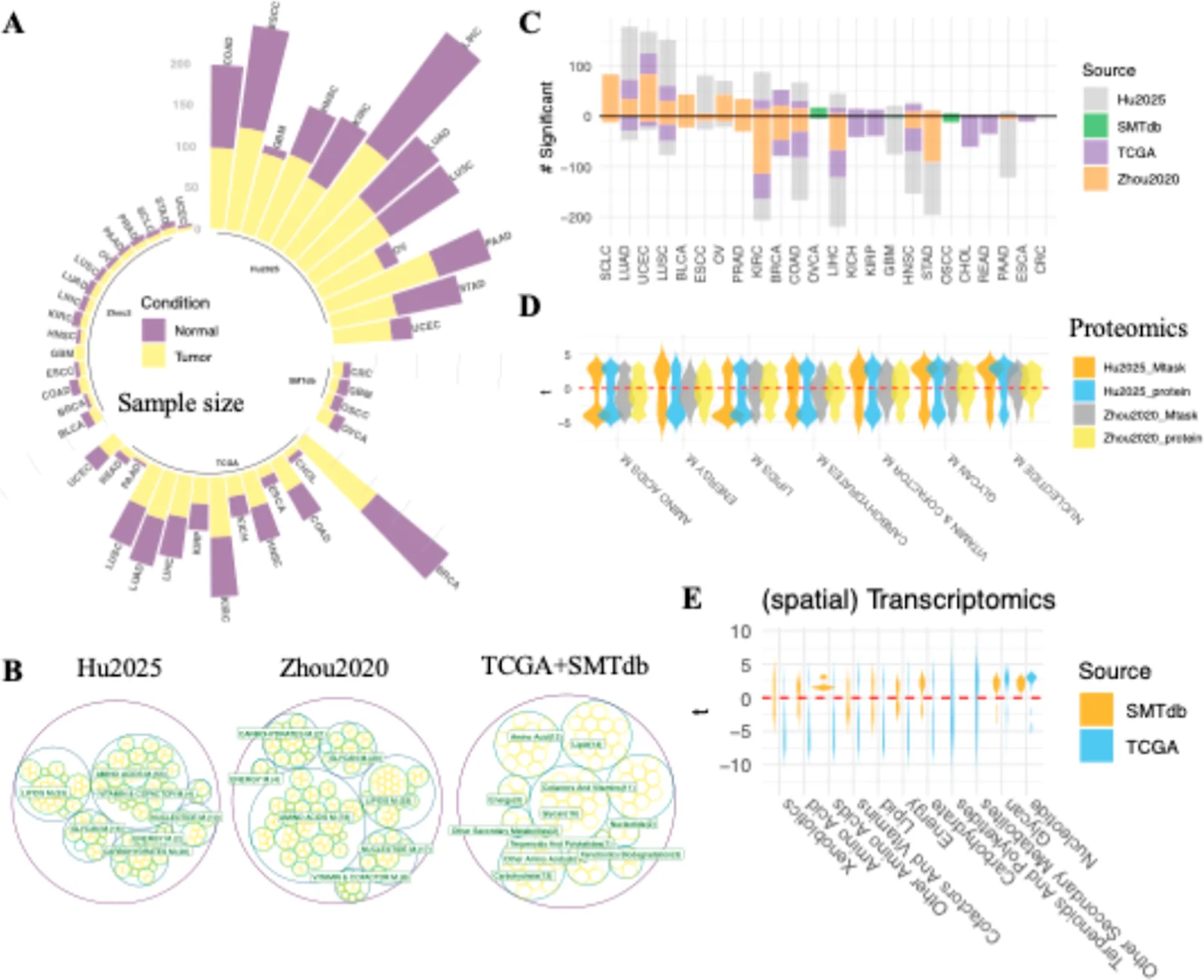
Pan-cancer metabolic dysregulation across independent data sets from distinct omics modalities. (A) Circular barplot showing sample sizes for the four independent pan-cancer data sets analyzed. (B) Circular packing plot displaying a hierarchical overview of the number of identified metabolic tasks or pathways mapped to major metabolic class across each data set. (C) Quantity of significantly altered CellFie tasks/KEGG pathways by data set. Statistical significance was evaluated at adj. *p* < 0.05 for Hu2025_CellFie and TCGA_KEGG. Thresholds were expanded to adj. *p* < 0.1 for Zhou2020_CellFie (small sample size constraint) and unadjusted *p* < 0.1 for SMTdb_KEGG. The choice of an unadjusted *p* < 0.1 threshold for the spatial transcriptomics (SMTdb) cohort was guided by both technical and biological considerations. In spatial data sets, the comparison between tumor spots and adjacent stromal spots is inherently constrained by the biological “field effect”,^37^ where neighboring nonmalignant cells exhibit transitional transcriptomic states altered by the nearby tumor. Consequently, metabolic differentials are subtle. Enforcing stringent false discovery rate (FDR) corrections would result in an overly conservative removal of biologically relevant, localized metabolic programs, and these spatial findings are, therefore, treated as exploratory. (D,E) Violin plots showing the distribution of moderated *t*-statistics derived from differential analyses comparing tumor versus control samples across each metabolic class. Statistics were calculated at the CellFie task/protein level for proteomics data sets (D: Hu2025 and Zhou2020) and at the KEGG pathway level for bulk/spatial transcriptomics data sets (E: TCGA and SMTdb) using the limma EB framework. Downregulated features are plotted as negative values.

Comparison of tumors with matched normal samples revealed widespread metabolic reprogramming across cancer types, with consistent patterns across independent data sets. Notably, uterine corpus endometrial carcinoma showed strong metabolic upregulation in both CellFie-based proteomics data, KEGG-based TCGA data (Figures 1C, 2, S2A), and KEGG-based Hu2025 proteomics data sets (Figure S3A), a trend potentially associated with the frequent activation of the insulin-phosphoinositide 3-kinase (PI3K) signaling and estrogen signaling pathways.^38^ In contrast, liver hepatocellular carcinoma (LIHC) consistently ranked among the most metabolically downregulated cancers (Figures 1C, 2, S2A, and S3), in line with a liver tissue metabolomics study showing that the majority of significantly altered metabolites were reduced in tumor tissue relative to matched adjacent liver.^39^ Crucially, sensitivity analyses indicated that the downregulation of LIHC was robust to variations in cancer purity (Figure S4). Together, these results underscore robust and reproducible pan-cancer metabolic alterations across diverse cohorts.

**Figure 2:**
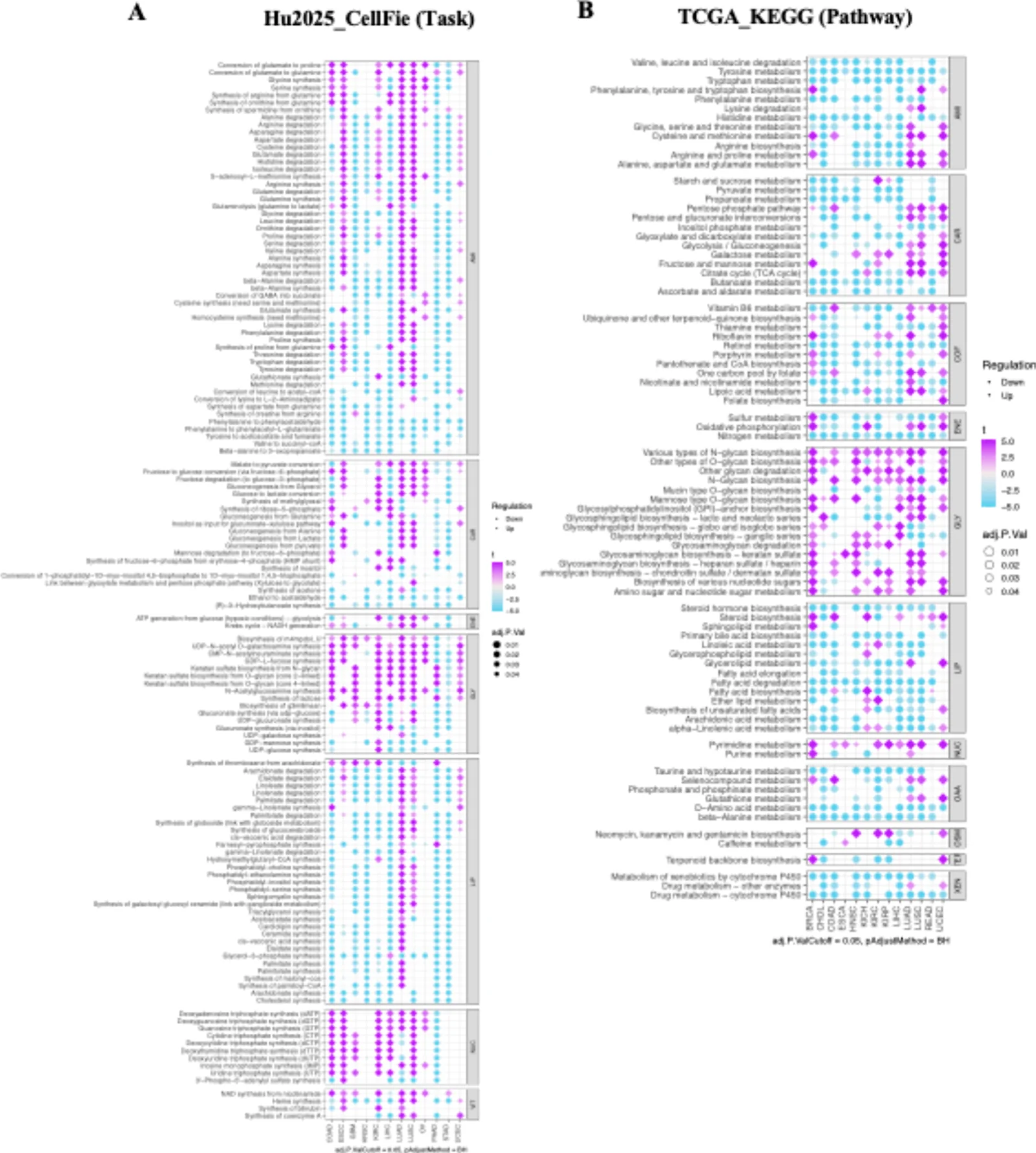
Pan-cancer metabolic dysregulation from distinct omics modalities. Dot plots showcasing both shared and distinct metabolic dysregulation profiles across cancer types derived from (A) Hu2025 proteomics data sets (CellFie-based tasks) and (B) TCGA transcriptomics data sets (KEGG-based pathways). Differential expression was evaluated by comparing primary tumor tissues against normal controls using the limma linear modeling framework. The color gradient corresponds to the moderated *t*-statistic, where positive values (purple) indicate upregulation in tumor tissue and negative values (blue) indicate downregulation. Dot size is proportional to statistical significance, scaled by −log_10_(adjusted *p*-value). To ensure readability and focus, data are filtered to display only significant results achieving an adj. *p* < 0.05.

Metabolic reprogramming in cancer showed significant heterogeneity across metabolic classes. Notably, nucleotide and glycan metabolism were consistently upregulated across all four data sets, with stronger effects observed in (spatial) transcriptomics (Figures 1D,E, S1T,U, Table S3). This upregulation was replicated at the protein level in the two proteomics data sets using paired differential analysis (Figure 1D, Table S3). Crucially, sensitivity analyses indicated that these metabolic patterns remained robust across variations in tumor purity, even though fewer pathways achieved significance (Figure S4B), highlighting the necessity of single-cell-level investigations. Multiomics integration via iNMF—restricted to glycan and nucleotide pathways—demonstrated a clear separation between malignant and normal tissues (Figure 3A,B). PERMANOVA analysis showed that this biological classification accounted for the dominant structural variance within the integrated space (*R̂* ^2^ = 0.211, *p* = 0.001), while confounding variations across data set layers were successfully mitigated, explaining a negligible fraction of the total variance (*R̂* ^2^ = 0.003, *p* = 0.002). Conversely, amino acid metabolism was among the most downregulated classes in three bulk omics comparisons (tumor vs matched normal), a trend that was not observed in our exploratory spatial omics analysis comparing malignant and stromal spots (Figures 1D,E, S1T–U). This divergence is likely attributed to two key factors: cellular dilution and cohort composition bias. First, bulk sequencing averages all cell types together, meaning that heavy infiltration of low-activity stromal and immune cells can potentially dilute and mask the hyper-active metabolic signatures localized strictly within malignant cells. Second, while the bulk data spans a comprehensive pan-cancer cohort, our spatial transcriptomic data set was restricted to four distinct cancer types with only a single overlapping malignancy between the two platforms (Figure 1A). Furthermore, the permissive statistical threshold (*p* < 0.1) utilized in the spatial analysis means these compartment-specific trends should be interpreted as exploratory rather than definitive.

**Figure 3:**
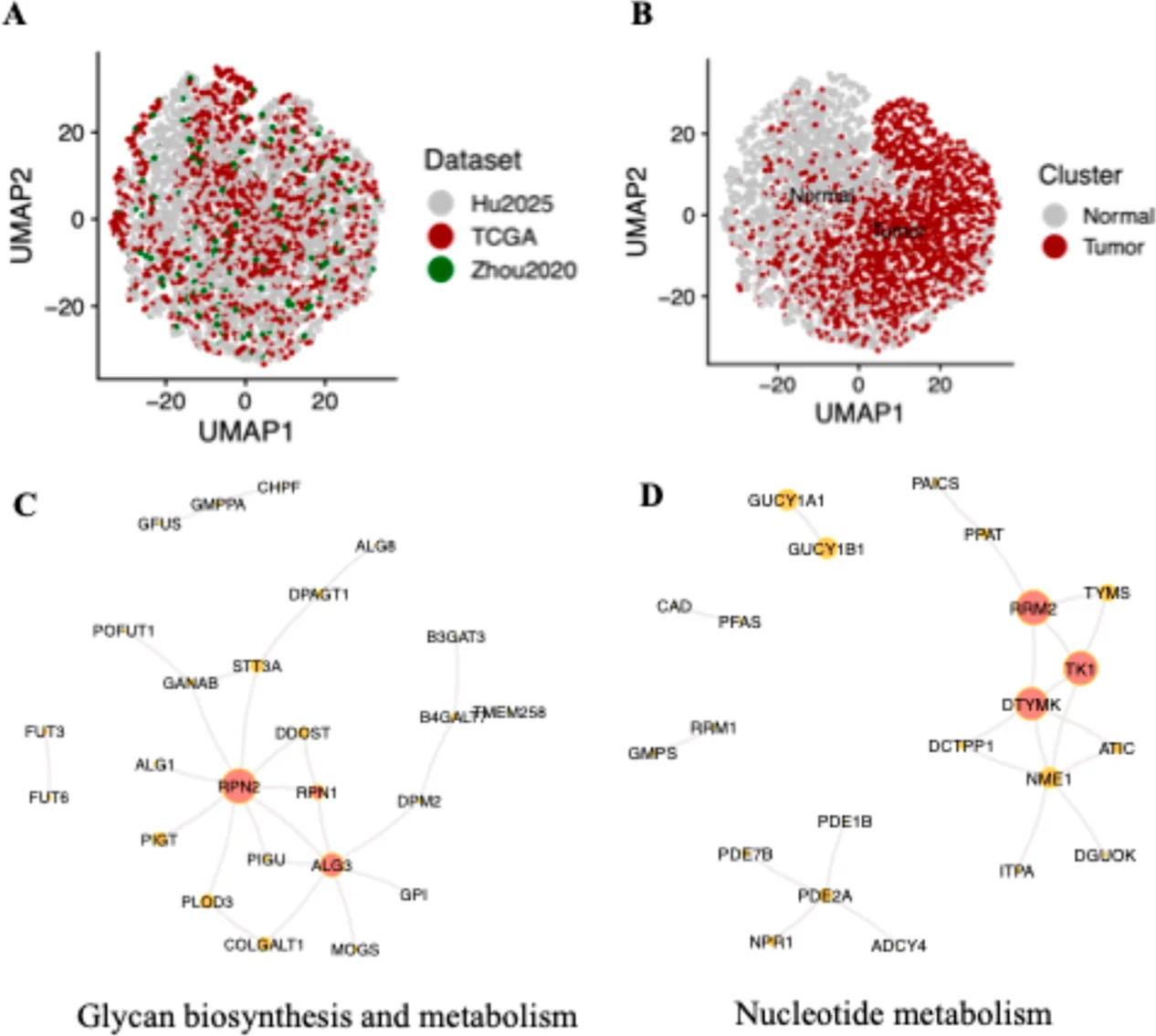
Multiomics integration and gene coexpression networks for glycan and nucleotide metabolism. (A,B) Integrative visualization of bulk proteomics (Hu2025 & Zhou2020) and bulk transcriptomics (TCGA) data sets based on glycan- and nucleotide-pathway metabolic scores. Scores were computed at the individual sample level using the GSVA algorithm applied to the KEGG pathways. (C,D) Gene coexpression networks for glycan and nucleotide metabolism constructed using igraph. Nodes represent individual genes, where node size is proportional to their normalized connectivity, and the top 1% most connected genes (≥3 per class) are highlighted as candidate network hubs (red). Network edges show strong coexpression relationships, defined by an absolute Spearman rank correlation coefficient >0.6, where edge widths are scaled by correlation strength. Gray edges indicate positive correlations, and blue edges indicate negative correlations. Networks were visualized using a force-directed Fruchterman–Reingold layout.

### The Tumor Glyco-switch: from Simple Sugars to Complex Capping

Among those broadly conserved alterations, glycan metabolism displayed a particularly coherent and biologically informative pattern. Tumors showed coordinated upregulation of pathways generating activated sugars for complex glycan modifications—including fucosylation and sialylation—accompanied by downregulation of common sugar nucleotide precursors such as UDP-glucose and GDP-mannose (Figures 2, S2–4; Table S3). Consistently, perturbations in mannose metabolism have been shown to disrupt nucleotide sugar biosynthesis and impair cancer cell proliferation.^40,41^ This shift is consistent with cancer-associated remodeling of cell surface, secreted, and extracellular matrix glycoproteins, which is frequently associated with enhanced invasiveness, immune evasion, and altered signaling through structurally complex glycans.^8,42^ The reduced synthesis of early sugar precursors likely reflects a metabolic prioritization toward downstream glycosylation pathways potentially linked to tumor survival and progression.

Biologically, increased CMP-*N*-acetylneuraminate and GDP l-fucose (GDP-l-fucose) synthesis (Figures 2A, S2A) are associated with the generation of sialylated and fucosylated glycans, which have been extensively linked to immune recognition and metastasis.^43,44^ Concurrently, the reduction in UDP-glucose and GDP-mannose production (Figures 2A and S2A) may indicate altered *N*-glycan core assembly or glycogen metabolism, further pointing toward a tumor-adapted metabolic state.

This robust, cross-cancer evidence—derived from a unified analytical framework—strengthens the concept that alterations in glycosylation pathways represent a broadly conserved metabolic hallmark of tumorigenesis. While previous studies have firmly established aberrant glycosylation as a hallmark of malignant cells,^45^ our systematic analysis provides a comprehensive, multidimensional characterization of these alterations across diverse cancers, highlighting these recurrent pathways as candidate frameworks for future diagnostic and therapeutic investigation.^46^

Gene coexpression network analysis in glycan metabolism identified key hub genes (*ALG3*, *RPN2*, and *RPN1*) that may be strongly associated with this metabolic rewiring, positioning them as high-priority candidates for future functional validation and therapeutic exploration (Figure 3C). Independent functional studies have experimentally validated all three central network hub genes as crucial oncogenic drivers (Table S4). Notably, ALG3, an ER-localized glycosylation enzyme, is implicated in promoting tumor growth and stemness, while driving resistance to both radiotherapy and immunotherapy.^47–49^ Similarly, RPN2, an oncogenic subunit of the *N*-oligosaccharyltransferase complex, is aberrantly expressed across multiple human cancers, where it promotes tumor progression, metastasis, and drug resistance.^50^

To evaluate the mathematical robustness of our network architecture, a formal sensitivity analysis was conducted by repeating the parameter optimization across three distinct scale-free topology constraints *R* ^2^ > 0.7, 0.8, and 0.9 and five top connectivity cutoffs (0.01, 0.05, 0.1, 0.2, and 0.3) for selecting hub genes. Strikingly, this sensitivity analysis revealed absolute structural convergence: all three *R* ^2^ thresholds selected the exact same underlying exponential powers, and the hub genes identified at the 1% threshold consistently maintained their position at the apex of the connectivity rankings across all cutoffs—with a minimum threshold of three hub genes enforced in cases where fewer were detected. These validations mathematically demonstrate that the identified hub features represent a resilient, high-confidence regulatory core that remains unchanged across variations in threshold stringency.

Multivariate survival analysis revealed that *ALG3*, *RPN2,* and *RPN1* consistently exhibited hazard ratios (HR) greater than 1.0 across the majority of evaluated cancer types, indicating that elevated expression of these hub genes might be generally associated with shorter overall survival (Figure S5A). However, after adjustment for clinical confounders, the prognostic impact of these genes was highly tissue-specific. High expression of *ALG3* was a significant independent risk factor exclusively in lung adenocarcinoma (*p* = 0.007), whereas *RPN2* upregulation was significantly tied to poor prognosis solely in Head and Neck Squamous Cell Carcinoma (HNSC) (*p* = 0.016) (Table S5).

Given the restricted, tissue-specific performance of standalone biomarkers, we sought to determine whether modeling these central features collectively via a multigene composite risk architecture would expand prognostic resolution and broaden predictive efficacy across diverse cancer types. Comparative evaluation demonstrated that the composite risk signatures systematically outperformed individual genetic markers by stabilizing effect-size estimations and expanding prognostic breadth: Individual constituent genes *ALG3*, *RPN2,* and *RPN1* exhibited highly volatile effect-size fluctuations across cancer types (HR range: 0.6–4.2) and retained independent significance in only two gene-cancer configurations (Table S5, Figure S5A). In contrast, the integrated multigene glycan risk score delivered highly stable risk stratification (HR range: 1.0–3.2) and achieved robust statistical association (*p* < 0.05) across five discrete cancer types (Table S5, Figure S5B). Thus, while individual hub genes yield fragile, localized prognostic signals, their strategic aggregation into a multigene composite framework captures a cumulative metabolic axis that bypasses the limitations of single-marker assessments.

### The Symmetrical Architecture of Tumor-Immune Metabolic Competition and Saturation

To map the functional interface between cell-autonomous metabolic dependencies and the microenvironment, we evaluated how individual pathway activity scores correlate with patterns of local immune infiltration. A strong positive association with estimated immune infiltration was predominantly clustered within specific glycan metabolic pathways (Figure S6). Conversely, central metabolic pathways—such as carbohydrate and amino acid metabolic pathways—exhibited a widespread trend toward weak, negative correlations with ESTIMATE immune scores (Figure S6).

Among the positive features, specialized glycan modules—specifically the biosynthesis of lacto/neolacto, globo/isoglobo, and ganglio series glycosphingolipids, as well as keratan and chondroitin/dermatan sulfates—are well-documented to participate in external cell-to-cell communication, vascular rolling, transendothelial migration, and the membrane organization and signaling of cell–surface immune checkpoint receptors.^51–55^ Consequently, the selective correlation of these glycan pathways with immune scores is unlikely to be an artifact of global metabolic acceleration. Instead, it is predicted to reflect the unique surface-remodeling requirements of the infiltrating host immune population. In contrast, the negative inclination in the correlations between baseline metabolic modules and immune scores is highly consistent with active metabolic competition and microenvironmental suppression within the tumor niche. Highly proliferative malignant cells and infiltrating, activated leukocytes aggressively compete for the same finite pools of local glucose and essential amino acids.^56^ Consequently, intense host immune pressure may potentially contribute to a localized nutrient depletion associated with decreased cell-autonomous central metabolic pathways.^57^

Crucially, projecting these immunometabolic signatures against tumor-versus-normal dysregulation scores revealed a striking divergence between baseline and specialized pathways, layered upon a highly heterogeneous pan-cancer background. For the core metabolic modules, which are predominantly downregulated in malignant tissue (Figures 1D,E and 2), we observed a trend of positive coupling between their tumor-specific suppression and their alignment with immune scores (Figure S6). This suggests that across heterogeneous cancer types, the severity of the tumor’s baseline metabolic shutdown closely reflects the intensity of local microenvironmental competition. In malignancies where central metabolic pathways are most severely suppressed relative to normal tissue, the negative correlation with immune scores is maximized, supporting the hypothesis that local immune infiltration compounding into these niches could potentially exacerbate nutrient depletion and core metabolic suppression.

In sharp contrast, specialized glycan pathways are predominantly upregulated in malignant tissue compared to matched normal controls (Figures 1D–E and 2). Intriguingly, these glycan modules exhibited a negative correlation between their tumor-versus-normal upregulation and their immunometabolic alignment with immune scores (Figure S6). This inverse relationship may point to a microenvironmental saturation effect: in cancer types where malignant cells natively hyper-activate glycan machinery, the bulk tissue baseline is saturated, masking immune-specific contributions. Conversely, in tumor contexts lacking intrinsic malignant glycan upregulation, these pathways become tightly synchronized with, and potentially reflective of, the influx of infiltrating host immune cells. Thus, glycan-associated surface remodeling may represent a context-dependent mechanism linked to the invading immune population, operating independently of the tumor’s native metabolic programming.

Notably, while these directional trends are robust, the absolute correlation coefficients remain largely moderate, with only 171 out of 1067 modules demonstrating a correlation magnitude equal to or greater than 0.5 (Table S6). This profile highlights a localized rather than global metabolic shift, underscoring the need for subsequent orthogonal validation—such as high-resolution single-cell or spatial transcriptomics^58^—to more precisely dissect cell-type-specific metabolic contributions from the bulk tissue background.

### Pan-Cancer Upregulation of Nucleotide Metabolism: A Conserved Hallmark

Moreover, nucleotide metabolism—covering the synthesis, salvage, and catabolism of purine and pyrimidine nucleotides—is a conserved metabolic program exploited by cancer cells, which has been extensively linked to proliferation, immune evasion, chemotherapy resistance, and metastasis.^59,60^ Leveraging a comprehensive pan-cancer analytical framework, we systematically quantify and characterize the upregulation of nucleotide metabolic pathways across a wide spectrum of tumor types. Our analysis indicates that this metabolic hallmark is not confined to isolated cancer subsets but represents a widespread feature observed in multiple malignancies, thereby expanding upon and unifying prior reports focused on individual tumor contexts.^61^

### Cross-Platform Evaluation and Correlation of CellFie Tasks and KEGG Pathways

To evaluate the consistency of our metabolic scoring, we correlated CellFie task scores against corresponding KEGG pathway enrichment scores in normal samples. High-level categorical alignment was highly consistent for glycan and nucleotide metabolism, demonstrating strong methodological alignment between our primary pan-cancer findings (Figure S7, Table S7). However, other metabolic sectors exhibited extensive cross-category correlation (Figure S7, Table S7). Notably, numerous CellFie tasks classified under lipid metabolism were strongly correlated with KEGG carbohydrate metabolism pathways, indicating an interconnected metabolic profile (Figure S7, Table S7). The discordance between high-level CellFie and KEGG classifications highlights the distinction between broad pathway boundaries and pinpointed operational capacity. While KEGG aggregates hundreds of genes into large, generalized maps based on static substrates, CellFie uses genome-scale metabolic models to evaluate smaller, highly specialized “tasks”.^23,26^ Consequently, the strong correlation between CellFie lipid tasks and KEGG carbohydrate pathways reflects network-wide functional crosstalk that large, compartmentalized KEGG boundaries inherently obscure.

### Contextualization Within Existing Pan-Cancer Literature and Methodological Advances

While foundational pan-cancer metabolic studies have successfully mapped broad oncogenic hallmarks, they have primarily relied on single-modality transcriptomic profiling and generic pathway gene-set enrichment analysis.^4,14^ These pioneering approaches often lack the resolution to trace specific reaction trajectories or separate tumor-intrinsic metabolic signals from microenvironmental noise. Our multiscale framework directly extends these prior efforts by introducing four critical methodological advances. First, we utilized the CellFie platform to calculate high-resolution metabolic task scores that are capable of tracing individual reaction trajectories. Second, we conducted our differential analysis directly at the pathway level, using tumor purity constraints as a rigorous sensitivity test to demonstrate that our core-identified metabolic dysregulation pattern remains robust against microenvironmental variations. Third, rather than filtering or screening components at the individual gene level, we grouped different pathways into broad metabolic classes and performed gene coexpression network modeling within each pathway class. This top-down systemic approach allowed us to map complete functional pathway topologies and pinpoint the specific structural hub genes (e.g., *ALG3*, *RPN1*, *RPN2*, *TK1*, *DTYMK*, and *RRM2*) that are centrally associated with this pan-cancer restructuring. We then performed multivariate Cox modeling directly on the extracted hub genes, both individually and collectively, to mathematically decouple their pure prognostic contributions from those of clinical covariates. Fourth, whereas traditional pipelines remain bound to computational estimation from blended bulk profiles, we evaluated our deconvolution framework using exploratory spatial transcriptomic data. By separating tissues into tumor and nontumor niches, we provide preliminary spatial support, indicating that our core metabolic dysregulation pattern is predominantly localized within the malignant niche rather than being an artifact of bulk tissue averaging. While these spatial insights are exploratory due to the relaxed statistical threshold (*p* < 0.1), they offer valuable contextual alignment for our bulk-derived model. Ultimately, this high-resolution approach highlights localized tissue architectures and suggests nonlinear microenvironmental saturation effects, setting a rigorous benchmark for isolating cell-autonomous metabolic dependencies across diverse oncological contexts.

### Beyond the Hallmark: The Heterogeneous Metabolic Architecture of Cancer

In contrast to the consistent upregulation observed in nucleotide and glycan metabolism, our analysis reveals that the dysregulation of other metabolic pathways exhibits substantial heterogeneity both between different cancer types and across independent data set collections. This variability may be attributed to a complex interplay of factors, including tissue-specific metabolic programming, diverse oncogenic mutations, the influence of the tumor microenvironment, and technical differences in sample processing and data generation.^3,62^

Tissue origin profoundly shapes the metabolic phenotype of tumors, as distinct organs possess unique baseline metabolic demands and enzyme expression profiles that tumors may retain or reprogram selectively.^63^ Furthermore, oncogenic drivers such as MYC proto-oncogene, Kirsten rat sarcoma viral oncogene homologue (KRAS), and tumor protein p53 mutations modulate metabolic pathways differently across cancer types, resulting in context-dependent metabolic adaptations.^2^ The tumor microenvironment, including nutrient availability, hypoxia, and stromal interactions, further sculpts metabolic reprogramming in a spatially and temporally dynamic manner.^64^ Technical heterogeneity across data sets—including differences in sample collection, sequencing platforms, and bioinformatic pipelines—can also contribute to observed variability.^65^ This pronounced metabolic heterogeneity challenges the identification of universal metabolic targets beyond the consistently conserved nucleotide and glycan pathways, emphasizing the value of integrative multicohort analyses to comprehensively capture metabolic diversity and enhance the robustness and predictive power of metabolic biomarkers.

## Conclusion

Our integrative analysis across proteomics, bulk transcriptomics, and spatial transcriptomics reveals conserved pan-cancer metabolic reprogramming, with coordinated upregulation of nucleotide and glycan metabolism. By implementing gene coexpression network modeling, we mapped the functional topology of these systems to isolate specific candidate regulators (hubs) associated with this pan-cancer metabolic restructuring. Furthermore, this high-resolution approach highlights a noticeable alignment between the estimated immune infiltration and specialized glycan pathways. Intriguingly, these glycan modules exhibit a distinct inverse relationship, where the magnitude of their tumor-intrinsic upregulation negatively correlates with their alignment with immune scores. This phenomenon suggests a potential microenvironmental saturation effect: in malignancies where tumor cells natively hyperactivate glycan machinery, the overall tissue baseline becomes saturated, thereby obscuring localized, immune-specific metabolic contributions in bulk molecular profiles. Ultimately, this framework provides a robust, data-driven roadmap to guide the future experimental validation of candidate therapeutic targets across diverse oncological contexts.

Despite the robustness of our multiomics discovery pipeline, certain inherent limitations regarding bulk transcriptomic resolution must be noted. Although our initial pathway-level purity constraints successfully isolated macroscopic tumor trends, bulk sequencing inherently averages expression profiles, which can mask subtle cell type-specific contributions. We addressed this directly by leveraging exploratory spatial transcriptomic data to separate tissues into tumor and nontumor niches. This spatial comparison indicated a trend wherein the core upregulation of glycan and nucleotide pathways is specifically enriched within the malignant niche, providing exploratory support for our bulk deconvolution adjustments and ensuring that the signal is not driven by surrounding nonmalignant cell types. However, because these compartment-specific insights were evaluated under a permissive statistical threshold (*p* < 0.1) and our current spatial cohort remains limited in both sample size and represented cancer types, future high-resolution validation across larger, multicancer spatial cohorts will be necessary to fully map these intratumoral metabolic boundaries across diverse oncological contexts.

## Supplementary Material















## Data Availability

All computational analyses were conducted using R (v4.4.3). To streamline the analytical workflow, we developed the parseMetab R package (v1.0.6), which facilitates metabolic activity inference from multiomics data, differential and network analyses, and cohort-level visualizations. The package source code is available on GitHub (github.com/Maj18/parseMetab), and a permanent archive of the specific version used is deposited on Zenodo (doi.org/10.5281/zenodo.21373531). To ensure platform-independent reproducibility and environment consistency, all analyses were executed within a dedicated Docker container (Docker Hub image ID: yuanli202004/cancer:v2.0.2). All underlying R scripts required to replicate the results and generate the figures are hosted at github.com/Maj18/cancer. The processed data sets supporting the conclusions of this study are provided within the article and its supplementary files.

## Notes

Figures S1–S8 provide a comprehensive overview of pan-cancer metabolic activity, dysregulation, gene-level characteristics, gene co-expression networks across distinct metabolic classes, hub-gene hazard-ratio profiles, immunometabolic coupling, and integrated cross-workflow metabolic network mapping, while Table S4 summarizes the functional validation profiles and experimental phenotypes of the identified hub genes (PDF)
Summary and sample information for pan-cancer data sets analyzed in this study (XLSX)
Metabolic activities inferred from proteomics, bulk transcriptomics, and spatial transcriptomics across pan-cancer data sets (XLSX)
Pan-cancer metabolic dysregulation results (XLSX)
Multivariate Cox regression summaries for individual hub genes and multigene composite risk scores (XLSX)
Immunometabolism (XLSX)
Cross-workflow metabolic correlations (XLSX)
