# Supplementary material for: Pan-Cancer Metabolic Landscapes: A Multiomics View
Source: J Proteome Res. 2026 Aug 27;25(10):5328–38. doi: 10.1021/acs.jproteome.6c00346 (PMC13640513; doi:10.1021/acs.jproteome.6c00346)
Supplement: Supplementary file 1 [file pr6c00346_si_001.pdf]

Supporting information

Pan-cancer metabolic landscapes: A multi-omics view

Yiguang Bai[1,2#*] Qiaoling Chen[3,4#] Yuan Li[5,6*]

[1]Department of Orthopedics, Beijing Anzhen Nanchong Hospital, Capital Medical University (Nanchong Central Hospital), Nanchong 637000, China

[2]Department of Orthopedics, Nanchong Central Hospital, The Second Clinical Medical College of North Sichuan Medical College, Nanchong 637000, China

[3]Department of Oncology, Beijing Anzhen Nanchong Hospital, Capital Medical University (Nanchong Central Hospital), Nanchong 637000, China

[4]Department of Oncology, Nanchong Central Hospital, The Second Clinical Medical College of North Sichuan Medical College, Nanchong 637000, China

[5]National Bioinformatics Infrastructure Sweden (NBIS), Science for Life Laboratory, Lund University, Lund, 22387, Sweden

[6]Department of Immunotechnology, Lund University, Medicon Village, Lund, 22387, Sweden

Table of Contents

- **Fig. S1.** Summary of metabolic activity, dysregulation and gene-level characteristics across pan-cancer datasets.
- **Fig. S2.** Pan-cancer metabolic dysregulation across independent pan-cancer datasets from two distinct omics modalities.
- **Fig. S3.** KEGG-based pan-cancer metabolic dysregulation across independent pan-cancer datasets from two proteomics collections.

- **Fig. S4.** Pan-cancer metabolic dysregulation across different TCGA cohorts, controlling for cancer purity.
- **Fig. S5.** Hazard ratio profiles of hub genes and integrated signatures across multiple cancer types.
- **Fig. S6.** Pan-cancer landscape of immunometabolic coupling.
- **Fig. S7.** Integrated multi-tiered cross-workflow metabolic profile network mapping.
- **Fig. S8.** Gene co-expression networks for different metabolic classes.
- **Table S4**. Functional validation profiles and experimental phenotypes of network hub genes.
- **Table S1**. Summary and sample information for pan-cancer datasets analyzed in this study. (xlsx)
- **Table S2**. Metabolic activities inferred from proteomics, bulk transcriptomics, and spatial transcriptomics across pan-cancer datasets. (xlsx)
- **Table S3**. Pan-cancer metabolic dysregulation results. (xlsx)
- **Table S5**. Multivariate Cox regression summaries for individual hub genes and multi-gene composite risk scores. (xlsx)
- **Table S6**. Immunometabolism. (xlsx)
- **Table S7**. Cross-workflow metabolic correlations. (xlsx)

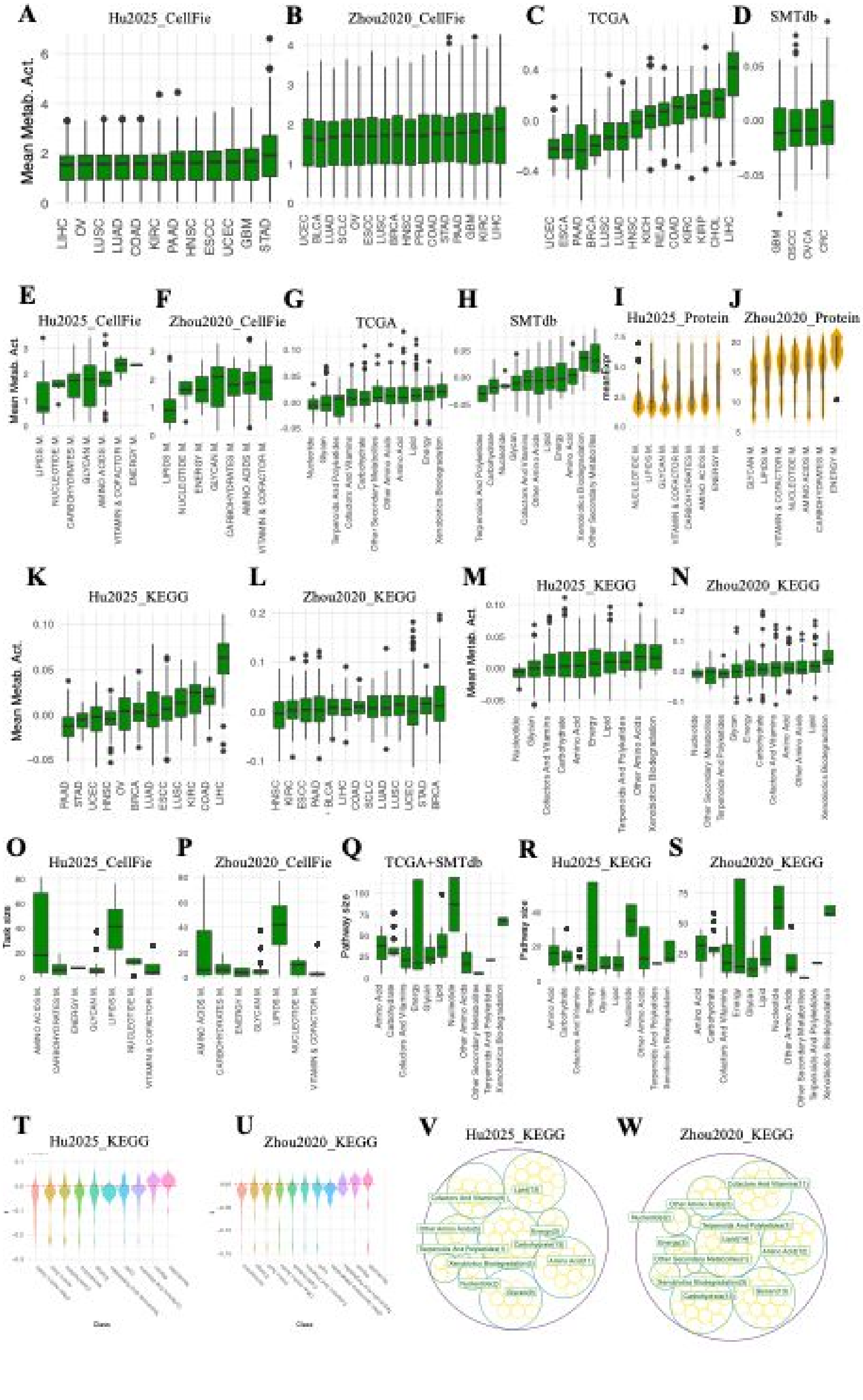

A
Hu2025_CellFie
B
Zhou2020_CellFie
C
TCGA
D
SMTdb
Mean Metab. Act.
E
Hu2025_CellFie
F
Zhou2020_CellFie
G
TCGA
H
SMTdb
I
Hu2025_Protein
J
Zhou2020_Protein
K
Hu2025_KEGG
L
Zhou2020_KEGG
M
Hu2025_KEGG
N
Zhou2020_KEGG
O
Hu2025_CellFie
P
Zhou2020_CellFie
Q
TCGA+SMTdb
R
Hu2025_KEGG
S
Zhou2020_KEGG
T
Hu2025_KEGG
U
Zhou2020_KEGG
V
Hu2025_KEGG
W
Zhou2020_KEGG

Fig. S1. Summary of metabolic activity, dysregulation and gene-level characteristics across pan-cancer datasets. (A-D, K-L) Boxplots showing mean metabolic activity per CellFie task/KEGG pathway for each cancer type, with one panel per dataset. (E-H, M-N) Boxplots showing mean metabolic activity per CellFie task/KEGG pathway for each metabolic class, with one panel per dataset. (I-J) Violin plots of mean protein expression for each metabolic class for the two proteomics datasets. (O-S) Boxplots of task/pathway size (number of genes per task/pathway) across metabolic classes for different datasets. (T-U) Violin plots showing the distribution of moderated *t* statistics from pathway-level differential analyses for each metabolic class in Hu2025_KEGG and Zhou2020_KEGG. (V-W) Overview of the number of identified metabolic pathways per metabolic class in Hu2025_KEGG and Zhou2020_KEGG.

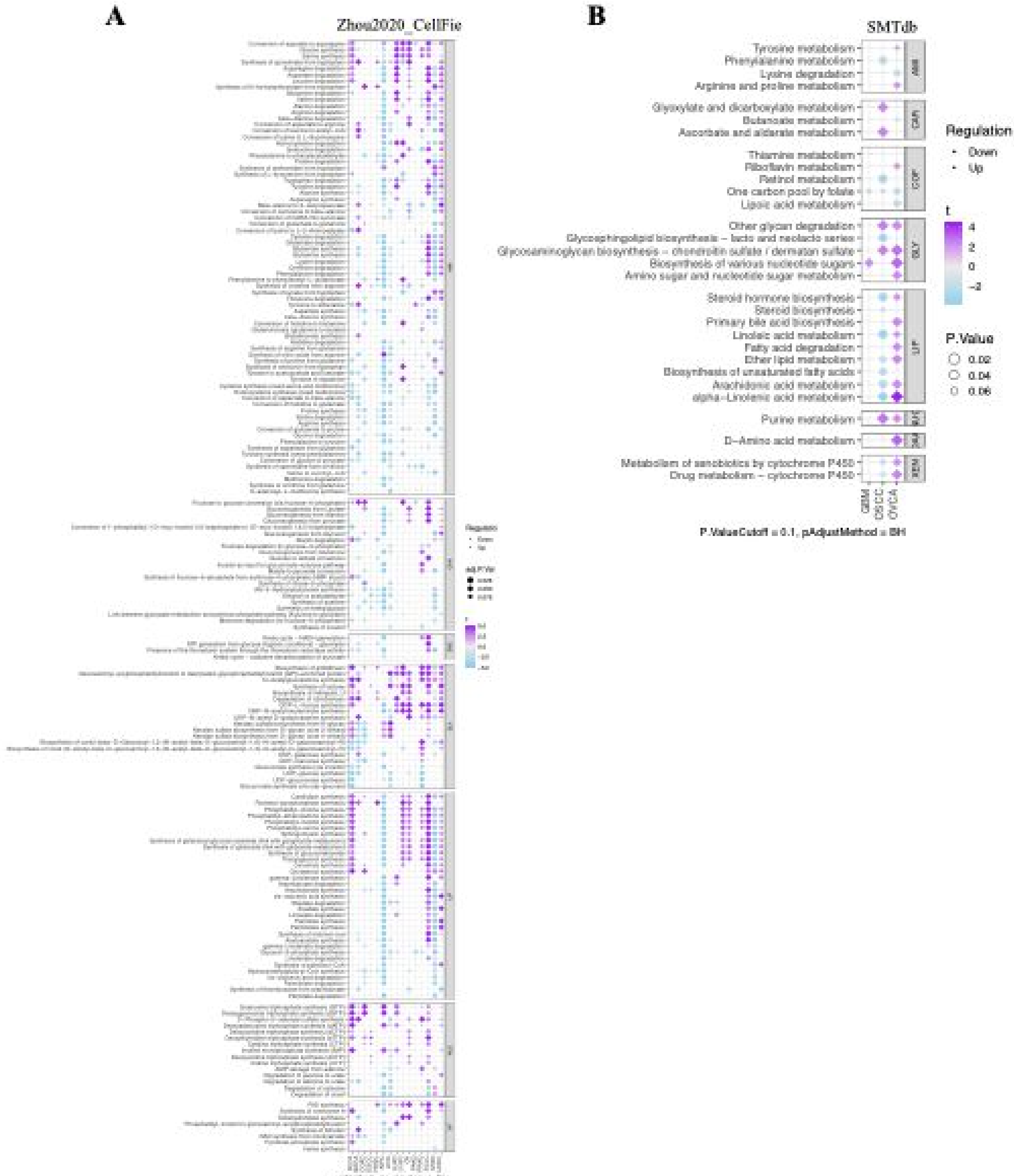

Fig. S2. Pan-cancer metabolic dysregulation across independent pan-cancer datasets from two distinct omics modalities. Dot plots of significant task/pathway-level dysregulation across cancer types in the Zhou2020 (A, adj. $p < 0.1$; CellFie tasks) and SMTdb (B, $p < 0.1$; KEGG pathways) data. Color represents moderated t statistics, and dot size reflects (adjusted) p values.

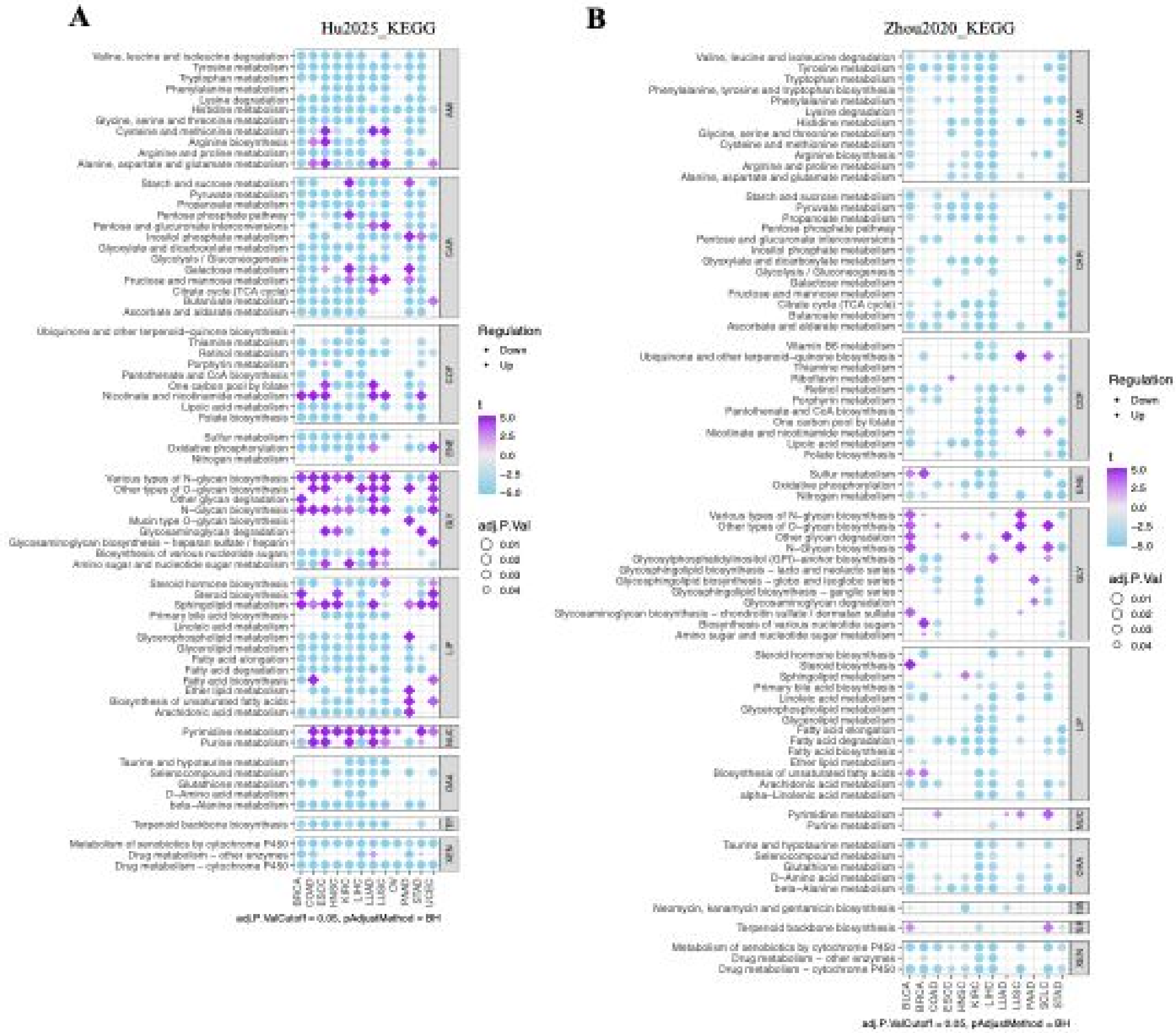

Fig. S3. KEGG-based pan-cancer metabolic dysregulation across independent pan-cancer datasets from two proteomics collections. Dot plots of significant pathway-level dysregulation across cancer types in the Hu2025_KEGG (A, adj. $p < 0.05$) and Zhou_KEGG (B, P. adj. $p < 0.1$ due to small sample sizes) data. Color represents moderated t statistics, and dot size reflects adjusted p values.

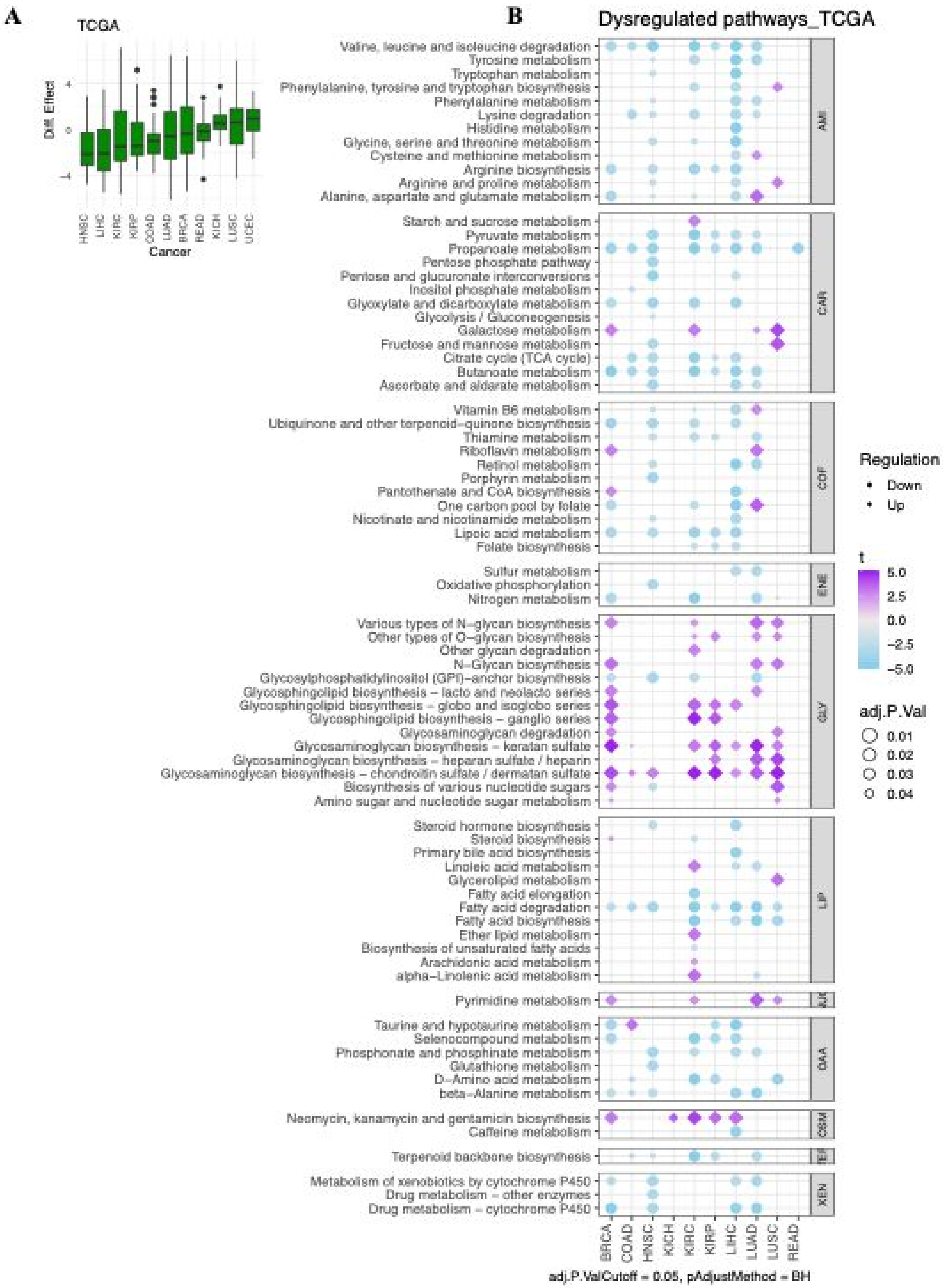

Fig. S4. Pan-cancer metabolic dysregulation across different TCGA cohorts, controlling for cancer purity. (A) Boxplot showing the distribution of differential effect sizes from KEGG pathway-level differential analyses across individual cancer types. (B) Dot plot of

KEGG metabolic pathway-level dysregulation across cancer types. Color represents moderated *t* statistics, and dot size reflects adjusted *p* values; only significant features (adj. $p < 0.05$) are shown.

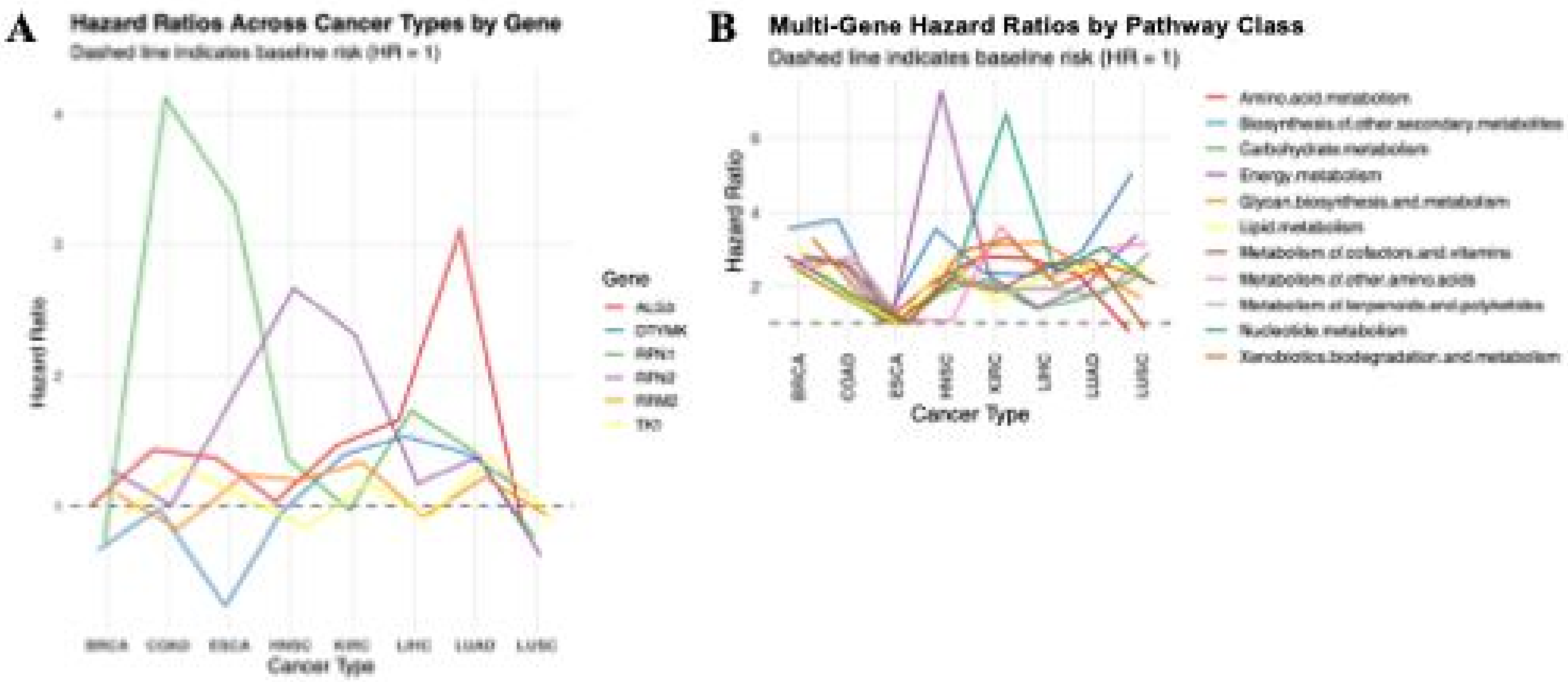

Fig. S5. Hazard ratio profiles of hub genes and integrated signatures across multiple cancer types. (A) The line plot displays the estimated hazard ratios (HR) for six target hub genes extracted from multivariate Cox proportional hazards models across different malignancies. The horizontal grey dashed line marks the baseline reference of HR = 1.0; data points situated above this baseline (HR > 1) denote an elevated risk of mortality associated with higher gene expression, while points below (HR < 1) represent a protective effect. Models are controlled for available patient clinical covariates, including age, pathologic disease stage, and gender. **(B)** Pan-cancer prognostic performance of the integrated multi-gene metabolic risk score. The plot displays the hazard ratios for the composite risk signatures across different cancer types, demonstrating the cumulative prognostic impact of the combined metabolic features. To ensure statistical reliability, only robust model estimates meeting a strict standard error threshold (SE < 5.0) are included.

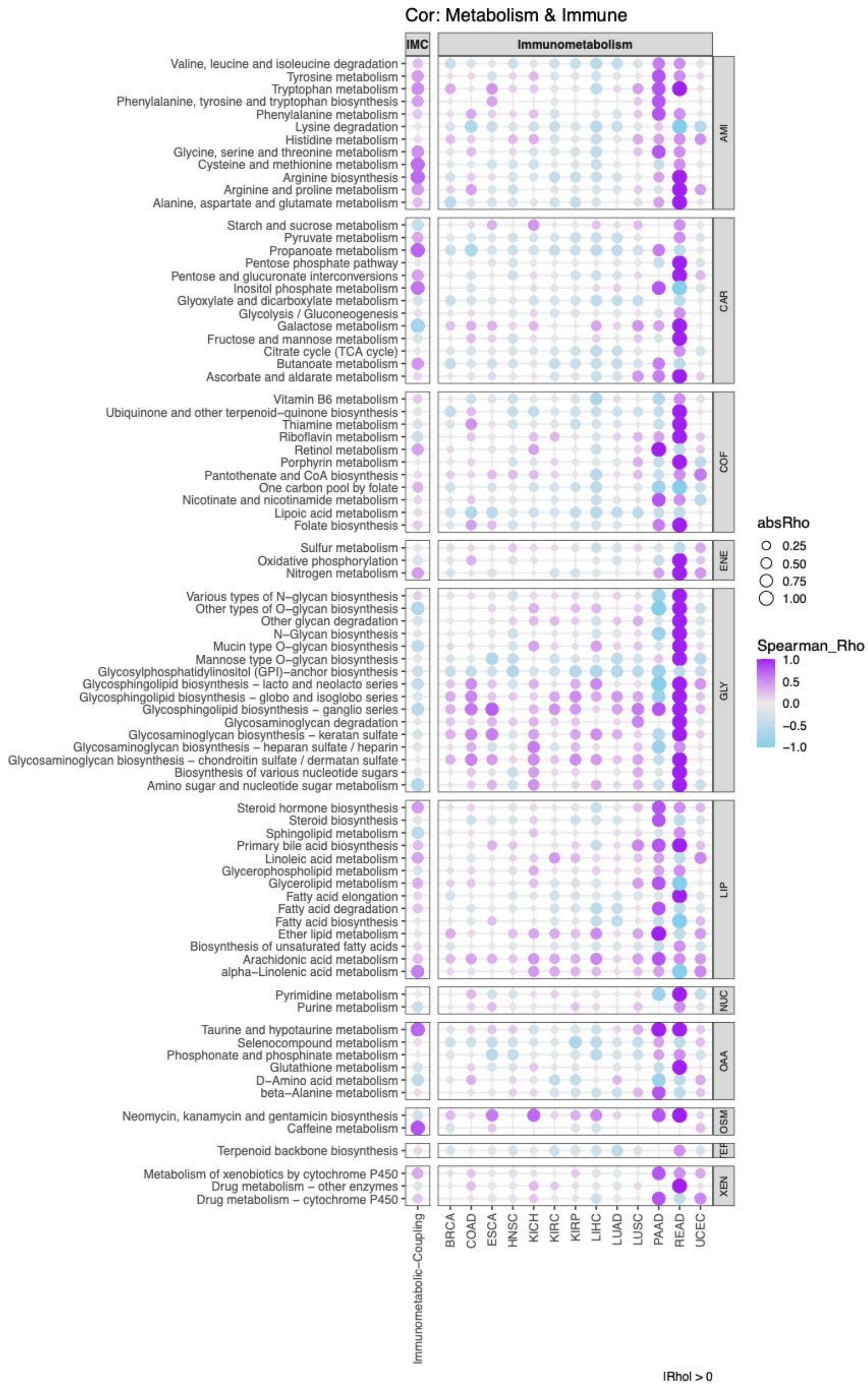

Fig. S6. Pan-cancer landscape of immunometabolic coupling. Dot plot illustrating the Spearman correlation (Immunometabolism) between metabolic GSVA enrichment scores and immune infiltration scores across diverse TCGA cancer types (Tumor tissues). The left panel displays the second-order correlation (Immunometabolic-coupling) between these correlation coefficients and overall tumor metabolic dysregulation (calculated as limma *t*-statistics

relative to normal tissue). In both panels, dot color represents the direction and strength of the correlation, while dot size scales with the absolute correlation coefficient value.

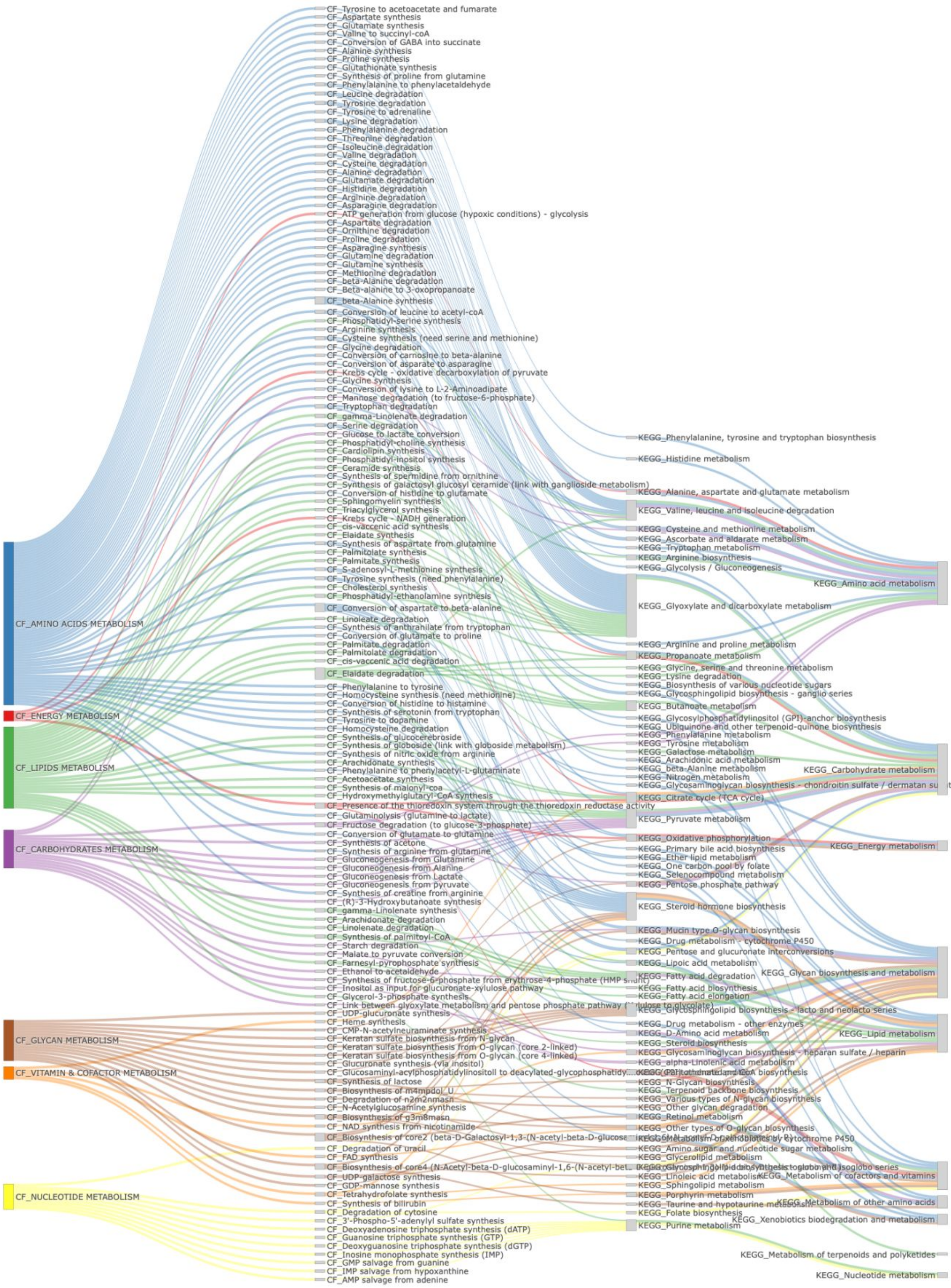

Fig. S7. Integrated multi-tiered cross-workflow metabolic profile network mapping. Network hierarchy spanning CellFie systems (Tier 1), CellFie tasks (Tier 2), KEGG pathways (Tier 3), and KEGG classes (Tier 4). Edge widths scale with the Spearman correlation coefficient (ρ). Known hierarchical links (Tiers 1-2 and Tiers 3-4) are

fixed at $\rho = 1.0$. Cross-workflow correlations (Tiers 2-3) were evaluated across cancer types in the Zhou2020 and Hu2025 datasets, retaining only high-stringency pairs with a correlation of median $\rho > 0.5$. The resulting data were merged into a consolidated union network by extracting the single strongest correlation for each unique CellFie task and KEGG pathway, removing redundant overlapping edges to minimize visual complexity.

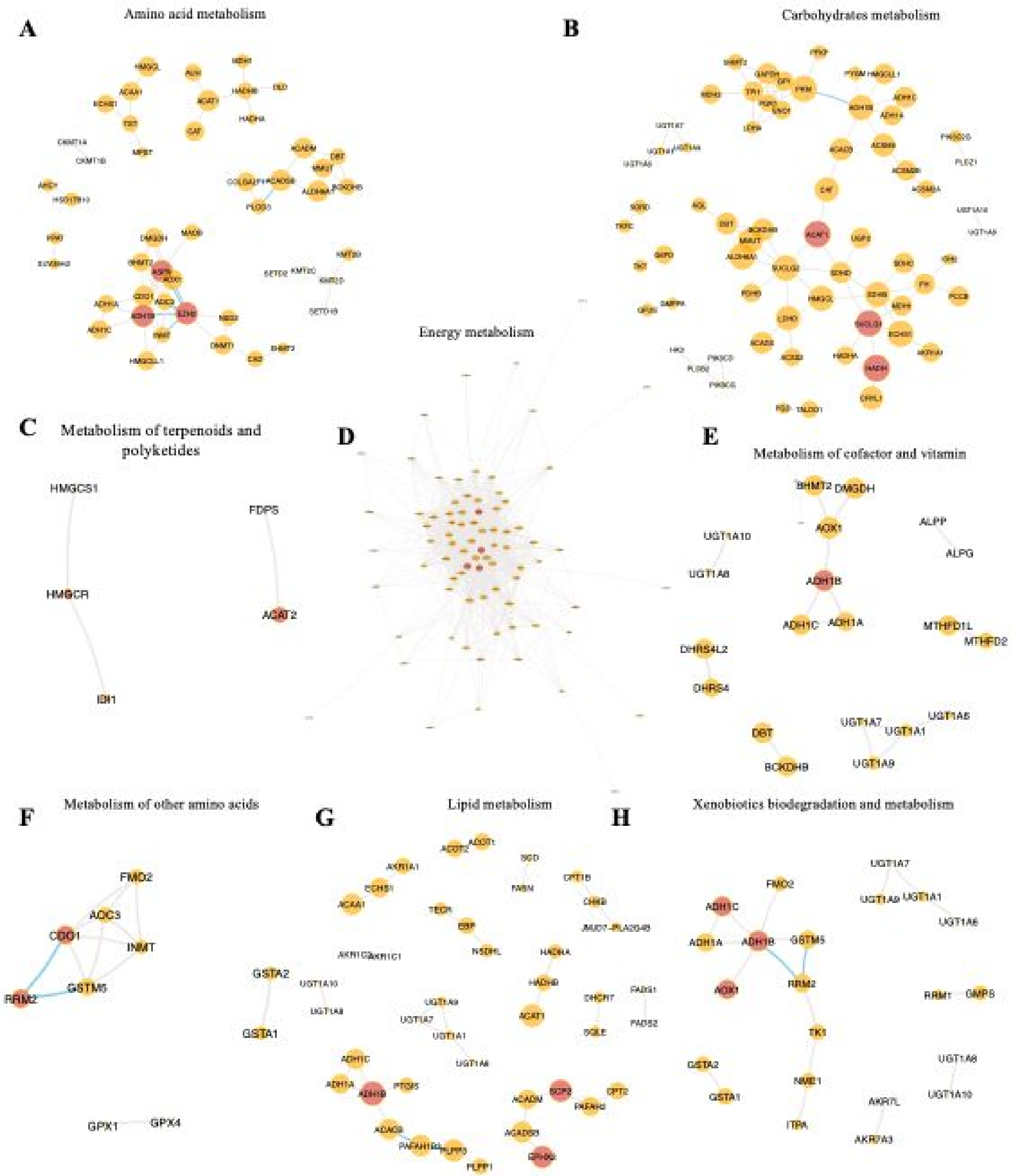

Fig. S8. Gene co-expression networks for different metabolic classes.
Networks nodes represent genes, sized by normalized connectivity, with the top 1% most connected genes (≥3 per class) highlighted as hubs (red). Edges show absolute correlation > 0.6, width scaled by correlation strength; grey edges indicate positive correlations and blue edges indicate negative correlations. Networks were visualized using a Fruchterman–Reingold layout.

Gene symbol	Function	Knockout/knockdown phenotype	Overexpression phenotype
ALG3	An ER-localized glycosylation enzyme	Inhibits caner cell growth and stemness, while enhancing radio- and immunotherapy sensitivity[1-3].	Enhances radioresistance and cancer stemness[2].
RPN2	An oncogenic subunit of the N-oligosaccharyltransferase complex	Reverses resistance to both standard chemotherapy and radiation, suppresses metastasis, and inhibits proliferation[4-8].	Enhances radioresistance[5].
RPN1	An essential structural subunit of the N-oligosaccharyltransferase complex.	Inhibits tumor progression, reduces PD-L1 expression, and induces apoptosis[9-11].	Drives immune evasion[9].
TK1	A key cytosol-localized enzyme in the pyrimidine salvage pathway.	Suppresses tumor growth and proliferation, inhibits metastatsis and epithelial-mesenchymal transition, and triggers cell death[12-14].	Promotes tumor proliferation[14].
DTYMK	A critical nucleotide enzyme executing the core step of the thymidylate pathway.	Inhibits proliferation, cell cycle progression, and invasion[15-16].	Promotes cancer cell proliferation[17].
RRM2	The rate-limiting small subunit of ribonucleotide reductase.	Suppresses cancer cell growth and migration, induces ferroptosis, and increases vulnerability to immunotherapy and DNA-damaging therapies[18-21].	Promotes cancer cell proliferation[21].

Table S4. **Functional validation profiles and experimental phenotypes of network hub genes.** This table summarizes the baseline biochemical functions and the corresponding in vitro and in vivo phenotypes resulting from knockout/knockdown (inhibition) or overexpression of the six key signature genes (*ALG3, RPN2, RPN1, TK1, DTYMK,* and *RRM2*).

References

1. Xue, X.; Feng, Q.; Hong, X.; Lin, Z.; Luo, Y.; Li, Y. et al. Comprehensive analysis of ALG3 in pan-cancer and validation of ALG3 as an onco-immunological biomarker in breast cancer. *Aging (Albany NY)*. 2024, 16(3), 2320.
2. Sun, X.; He, Z.; Guo, L.; Wang, C.; Lin, C.; Ye, L. et al. ALG3 contributes to stemness and radioresistance through regulating glycosylation of TGF-β receptor II in breast cancer. *J. Exp. Clin. Cancer Res*. 2021, 40(1), 149.
3. Liu, P.; Lin, C.; Liu, Z.; Zhu, C.; Lin, Z.; Xu, D. et al. Inhibition of ALG3 stimulates cancer cell immunogenic ferroptosis to potentiate immunotherapy. *Cell. Mol. Life Sci*. 2022, 79(7), 352.
4. Bi, C.; Jiang, B. Downregulation of RPN2 induces apoptosis and inhibits migration and invasion in colon carcinoma. *Oncology reports*. 2018, 40(1), 283-293.
5. Li, C.; Ran, H.; Song, S.; Liu, W.; Zou, W.; Jiang, B. et al. Overexpression of RPN2 suppresses radiosensitivity of glioma cells by activating STAT3 signal transduction. *Mol. Med*. 2020, 26(1), 43.
6. Han, C.; Chen, S.; Ma, H.; Wen, X.; Wang, Z.; Xu, Y. et al. RPN2 predicts poor prognosis and promotes bladder cancer growth and metastasis via the PI3K-Akt pathway. *OncoTargets Ther*. 2021, 1643-1657.
7. Fujiwara, T., Takahashi, R. U., Kosaka, N., Nezu, Y., Kawai, A., Ozaki, T., & Ochiya, T. (2014). RPN2 gene confers osteosarcoma cell malignant phenotypes and determines clinical prognosis. *Mol. Ther. Nucleic Acids*, 3.

8. Li, C.; Ran, H.; Song, S.; Liu, W.; Zou, W.; Jiang, B. et al. Overexpression of RPN2 suppresses radiosensitivity of glioma cells by activating STAT3 signal transduction. *Mol. Med*. 2020, 26(1), 43.
9. Lin, S.; Wu, C.; Liu, Q.; Luo, F.; Tan, J. RPN1 Is Associated With Immunosuppression in Pan-Cancer and Affects the Malignant Phenotype of Tumor. *FASEB J*. 2025, 39(16), e70978.
10. Shen, W. J.; Zhang, Y. RPN1 promotes the proliferation and invasion of breast cancer cells by activating the PI3K/AKT/mTOR signaling pathway. *Discov. Oncol*. 2024, 15(1), 25.
11. Luo, P.; Li, Z.; He, H.; Tang, Y.; Zeng, L.; Luo, L. et al. Exploring the impact of RPN1 on tumorigenesis and immune response in cancer. *FASEB J*. 2025, 39(6), e70345.
12. Sun, Y.; Zhang, K.; Wang, T.; Zhao, S.; Gao, C.; Xue, F. et al. A comprehensive analysis and experimental validation of TK1 in uterine corpus endometrial carcinoma. *Sci. Rep*. 2024, 14(1), 6134.
13. Liu, C.; Wang, J.; Zhao, L.; He, H.; Zhao, P.; Peng, Z. et al. Knockdown of thymidine kinase 1 suppresses cell proliferation, invasion, migration, and epithelial–mesenchymal transition in thyroid carcinoma cells. *Front. Oncol*. 2020, 9, 1475.
14. Li, Z.; Wu, Z.; You, X.; Tang, N. Pan-cancer analysis reveals that TK1 promotes tumor progression by mediating cell proliferation and Th2 cell polarization. *Cancer Cell Int*. 2024, 24(1), 329.
15. Zheng, J.; Wang, Q.; Yan, L.; Pan, Q.; Chen, X.; Chen, Q. The Biological Behavior and Clinical Application Prospects of Deoxythymidine Kinase Gene in Tumors. *Technol. Cancer Res. Treat*. 2024, 23, 15330338241265396.

16. Oziębło, S.; Mizera, J.; Górska, A.; Krzyziński, M.; Karpiński, P.; Markiewicz; A. et al. Co-targeting of DTYMK and PARP1 as a potential therapeutic approach in uveal melanoma. *Cells*. 2024, 13(16), 1348.
17. Liu, Y.; Marks, K.; Cowley, G.S.; Carretero, J.; Liu, Q.; Nieland, T.J.; Xu, C.; Cohoon, T.J.; Gao, P.; Zhang, Y.; et al. Metabolic and functional genomic studies identify deoxythymidylate kinase as a target in LKB1-mutant lung cancer. Cancer Discov. 2013, 3, 870–879.
18. Tang, B.; Xu, W.; Wang, Y.; Zhu, J.; Wang, H.; Tu, J. et al. Identification of critical ferroptosis regulators in lung adenocarcinoma that RRM2 facilitates tumor immune infiltration by inhibiting ferroptotic death. *Clin. Immunol*. 2021, 232, 108872.
19. Xiong, W.; Zhang, B.; Yu, H.; Zhu, L.; Yi, L.; Jin, X. RRM2 regulates sensitivity to sunitinib and PD-1 blockade in renal cancer by stabilizing ANXA1 and activating the AKT pathway. *Adv. Sci*. 2021, 8(18), 2100881.
20. Xing, H.; Cai, S.; Wu, C.; Yang, W.; Yang, A.; Zhang, X. et al. Ribonucleotide reductase regulatory subunit M2 as a macromolecular target bridging small cell lung cancer progression and SARS-CoV-2 infection. *Int. J. Biol. Macromol*. 2026, 152479.
21. Nunes, C.; Depestel, L.; Mus, L.; Keller, K. M.; Delhaye, L.; Louwagie, A. et al. RRM2 enhances MYCN-driven neuroblastoma formation and acts as a synergistic target with CHK1 inhibition. *Sci. Adv*. 2022, 8(28), eabn1382.
